# Fernblock Prevents Dermal Cell Damage Induced by Visible and Infrared A Radiation

**DOI:** 10.3390/ijms19082250

**Published:** 2018-08-01

**Authors:** Alicia Zamarrón, Silvia Lorrio, Salvador González, Ángeles Juarranz

**Affiliations:** 1 Department of Biology, Faculty of Sciences, Autónoma University of Madrid, IRYCIS, 28049 Madrid, Spain; aliszm@gmail.com (A.Z.); silvia.lorrio@gmail.com (S.L.); 2 Department of Medicine and Medical Specialties, Alcalá de Henares University, 28805 Madrid, Spain; salvagonrod@gmail.com

**Keywords:** photoaging, natural extract, in vitro studies, dermal fibroblast, extracellular matrix

## Abstract

Sun overexposure leads to higher risk of photoaging and skin cancer. The contribution of infrared (IR) and visible light (VIS) radiation is currently being taken into account in their pathogenesis. Erythema, hyperpigmentation, genotoxicity or the increase of matrix metalloproteinases (MMPs) expression are some of the effects induced by these types of radiation. Extracts of various botanicals endowed with antioxidant activity are emerging as new photoprotective compounds. A natural extract from *Polypodium leucotomos* (Fernblock^®^, FB) has antioxidant and photoprotective properties and exhibits a strong anti-aging effect. In this study, we evaluated the protective capacity of FB against the detrimental effects of infrared A (IRA) and VIS radiation in human dermal fibroblasts. We analyzed the effects of FB on the morphology, viability, cell cycle and expression of extracellular matrix components of fibroblasts subjected to VIS and IRA. Our results indicate that FB prevents cell damage caused by VIS and IRA. Moreover, it reduces the increase in MMP-1 and cathepsin K expression induced by both VIS and IRA radiation, and curbs alterations in fibrillin 1, fibrillin 2 and elastin expression. All these findings support FB as a feasible approach to prevent or treat skin damage caused by IRA or VIS exposure.

## 1. Introduction

Skin aging is a complex biological process characterized by a progressive loss of its physiological integrity. It is influenced by both intrinsic factors (such as genetic, metabolic or hormone-related) and extrinsic factors (such as exposure to environmental pollutants or radiation of specific wavelengths). This is a time-dependent process in which accumulation of cellular damage is postulated as the cause of aging. Simultaneously, cellular damage may occasionally provide aberrant advantages to certain cells and promote cancer. In general, cancer and aging appear as two different manifestations of the accumulation of cellular damage [1,2,3].

Sun radiation is a major cause of photoaging and photocarcinogenesis. Sunlight contains radiation of many different wavelengths, some of which traverse the atmosphere and reach ground level. Of these, ultraviolet (UV) light is the major cause of photoaging and photocarcinogenesis. However, infrared (IR) and visible light (VIS) radiation may also contribute to the pathogenesis of these processes [4,5].

Human skin is exposed to IR from several natural as well as artificial sources. IR radiation is likely to exert biologic effects on human skin. Clinical reports have described its ability to cause and enhance actinic skin damage [5]. The IR spectral region is divided into three sub regions: IR-A (760–1400 nm), IR-B (1400–3000 nm) and IR-C (3000 nm–1 mm). The largest part of solar radiation is IR, which reaches the subcutaneous tissue without markedly increasing the skin surface temperature. This is due to the filtering of the sun radiation by water vapor in the atmosphere of the earth. The filter effect of water decreases most parts of IR-B and IR-C and the absorption bands of water within IR-A that reach the earth’s surface. It is therefore important to take into account ground-based conditions and solar irradiance measurements rather than satellite measurements to study solar radiation effects on the skin [6]. Technically, water-filtered IR-A (wIRA) is produced by special radiators, whose full spectrum of radiation is passed through a cuvette containing water, in order to reproduce the conditions of solar IR skin irradiation at the earth’s surface level [7]. It has been reported that prolonged exposure to IR-A radiation leads to erythema, reticular hyperpigmentation and telangiectasia accompanied by histological alterations such as modifications of dermal fibers [8]. Moreover, at a molecular level, IR exposure results in increased expression of matrix metalloproteinases (MMPs), endopeptidases responsible for the degradation of extracellular matrix (ECM) components such as collagen and elastin. These fibrillary proteins support the normal structure of the skin and their reorganization underlies wound healing, angiogenesis and cutaneous photoaging [9]. Mitochondrial reactive oxygen species (ROS) generation was shown to be the initiating event and to induce increased transcription and translation of the MMP-1 gene via activation of the Mitogen-Activated Protein Kinases (MAPKs) pathway [4,5,10,11,12].

Although sunlight at the earth’s surface level is comprised of up to 44% visible light (VIS, 400–700 nm), few studies have sought to determine its cutaneous effects. In 1962, Pathak et al. [13] reported that exposure to VIS causes immediate pigment darkening (IPD), which is a transitory darkening of skin that can become persistent (persistent pigment darkening, PPD). VIS exerts various biological effects including erythema, pigmentation, thermal damage and free radical production. VIS also promotes indirect DNA damage through the generation of (ROS) [14]. In 2008, Botta et al. [15] reported that VIS induced genotoxic effects in human keratinocytes through oxidative stress mechanisms similar to those induced by UV light. Furthermore, a number of photodermatoses have an action spectrum in the visible light range, even though most of the currently available sunscreens offer weak, if any, protection against VIS [14,15,16].

The search for natural compounds that improve and stimulate the regeneration of the skin constitutes a crucial objective in dermatology, together with sun education [17]. *Polypodium leucotomos* (PL) is a fern from Central America that has been traditionally used for the treatment of inflammatory skin disorders such as psoriasis or atopic dermatitis. Fernblock (FB) is a natural extract from PL leaves endowed with antioxidant, photoprotective and immunoregulatory properties which is able to directly prevent DNA mutations in skin cells. This extract exhibits a strong anti-aging effect as it prevents the morphological effects caused by increased oxidative stress. Additional anti-aging effects include inhibition of MMPs expression and increased tissue inhibitors of metalloproteases (TIMPs) expression. Moreover, FB inhibits activator protein 1 (AP-1) and nuclear factor kappa-light-chain-enhancer of activated B cells (NFκB) transcription, and cyclooxygenase-2 (COX-2) expression induced by UV radiation [18,19,20]. Likewise, FB increases the secretion of elastin, a dermal component that plays an important role in cutaneous elasticity and retraction [9,21]. Fibrillins 1 and 2 are the main constituents of extracellular microfibrils responsible for the biomechanical properties of most tissues and organs, and they also play an important role in the assembly of elastin in elastic fibers formation [22]. Cathepsin K (CTSK), a lysosomal cysteine protease with strong collagenolytic activity, has been described to be highly expressed in skin fibroblasts under certain pathologic conditions, such as scarring or inflammation [23,24].

In this study, we have evaluated the protective capacity of FB against the detrimental effects induced by IR (specifically wIRA) and VIS radiation in human dermal fibroblasts. We report that FB prevents the changes induced by wIRA and VIS on the morphology, viability and cell cycle of human fibroblasts, as well as on the expression of different ECM components including MMP-1, CTSK, fibrillins 1 and 2 and elastin.

## 2. Results

### 2.1. Effects of VIS/wIRA Radiation and FP+VIS/wIRA Treatment on Fibroblast Morphology and Survival

**Figure 1:**
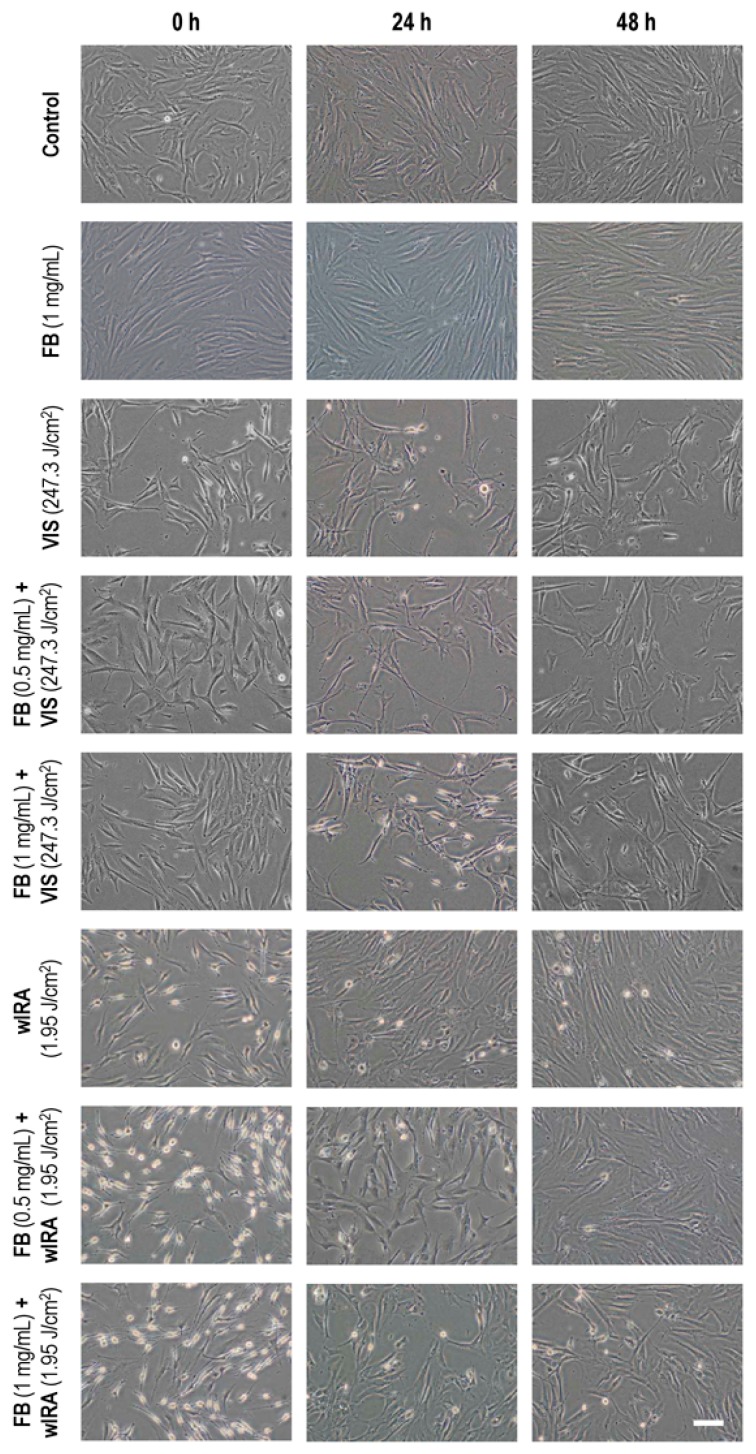
Effects of visible light (VIS), Fernblock (FB)+VIS, water-filtered IR-A (wIRA) and FB+wIRA on cellular morphology. Cells were subjected to FB (1 mg/mL), VIS light (247.3 J/cm^2^), wIRA light (1.95 J/cm^2^), FB+VIS or FB+wIRA treatment. Cultures were observed 0, 24 and 48 h after treatments and images were captured at these different time points. Scale bar: 100 µm.

To evaluate the effects of VIS or wIRA light alone and in combination with FB pre-treatment on cell morphology, cultures were observed in vivo 0, 24 and 48 h after treatment (Figure 1). Incubation with FB 1 mg/mL did not cause any change in fibroblast morphology as compared to control cultures. Immediately after VIS radiation (247.3 J/cm^2^), cultures showed cytoplasmic retraction, long cytoplasmic projections and cellular stretching, all signs of cellular stress. These alterations remained for up to 48 h after light exposure. Cells subjected to FB+VIS treatment also presented the described morphological changes, although they became more evident 24 h after irradiation. Phase contrast images in Figure 1 showed that wIRA light (1.95 J/cm^2^) caused cytoplasmic retraction and the appearance of rounded morphologies. These alterations were more obvious immediately after irradiation but almost disappeared 48 h after light exposure. When cells were treated with FB for 24 h prior to irradiation, the same morphological changes were observed with a modest increase in the number of rounded cells. As in the case of wIRA alone, alterations also disappeared 48 h after FB+wIRA.

**Figure 2:**
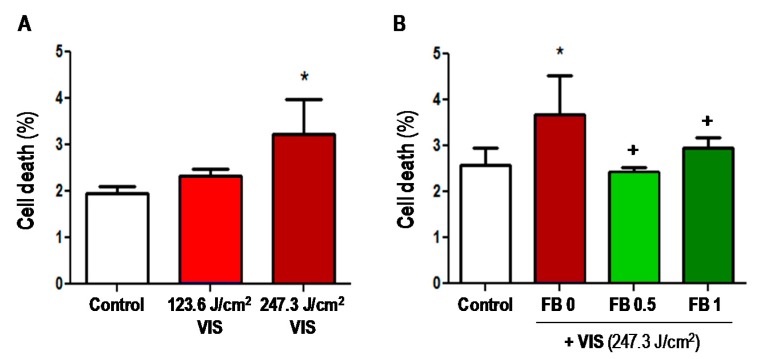
Effects of VIS and FB+VIS on cell survival. (**A**) Cells were subjected to 123.6 or 247.3 J/cm^2^ of VIS radiation. Cell death was measured by fluorescent-activated cell sorting (FACS) 24 h after treatments; (**B**) cells were incubated with FB (0.5 or 1 mg/mL) for 24 h and then were exposed to VIS light (247.3 J/cm^2^). Cell death was determined by FACS 24 h after treatments. (* *p* ≤ 0.05, with respect to Control; ^+^ *p* ≤ 0.05, with respect to FB 0).

The effect of VIS with and without FB pre-treatment on cell viability was quantified by flow cytometry (fluorescent-activated cell sorting, FACS) (Figure 2). In all cases, cell death rates after treatments ranged below 5%. Cell death rate was significantly increased after 247.3 J/cm^2^ of VIS as compared to the control (Figure 2A); however, pre-treatment with FB (0.5 or 1 mg/mL) prior to VIS light, diminished cell death rate approximately to control levels and was significantly lower than in non-pre-treated cells (*p* ≤ 0.05) (Figure 2B). Therefore, FB appears to prevent the cell damage caused by VIS light.

**Figure 3:**
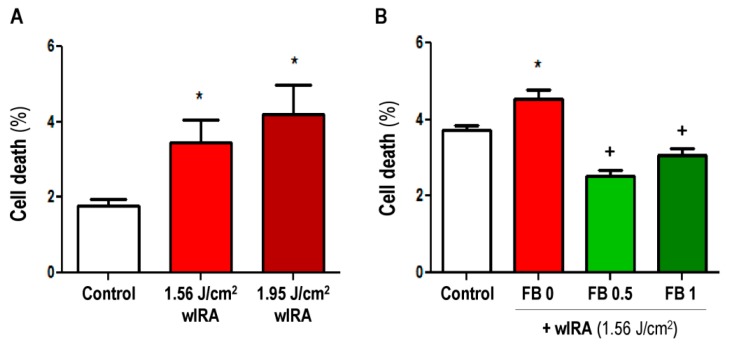
Effects of wIRA and FB+wIRA on cell survival. (**A**) Cells were exposed to 1.56 or 1.95 J/cm^2^ of wIRA. Cell death was measured by FACS 24 h after treatments; (**B**) cells were incubated with FB (0.5 or 1 mg/mL) for 24 h and then were exposed to wIRA light (1.56 J/cm^2^). Cell death was determined by FACS 24 h after treatments. (* *p* ≤ 0.05, with respect to Control; ^+^ *p* ≤ 0.05, with respect to FB 0).

Likewise, we quantified the effect of wIRA with and without FB pre-treatment on cell viability (Figure 3). Basal cell death was similar to the experiments shown in Figure 3. wIRA light (1.56 and 1.95 J/cm^2^) induced a significant dose-dependent increase in cell death percentage with respect to control (non-irradiated) cells (*p* ≤ 0.05) (Figure 3A). However, treatment with 0.5 or 1 mg/mL FB before irradiation significantly reduced cell death rate (Figure 3B) with respect to non-irradiated cells (*p* ≤ 0.05). This suggests that although it did not eliminate morphological alterations, FB can prevent wIRA-induced cell damage.

### 2.2. Effects of VIS/wIRA Light and FB+VIS/wIRA Treatment on the Cell Cycle

**Figure 4:**
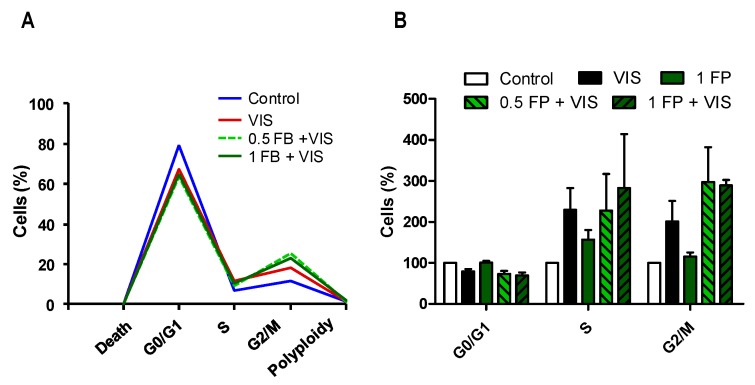
Effects of VIS and FB+VIS on cell cycle. Cell cycle was evaluated 24 h after the indicated treatments by flow cytometry. (**A**) Percentage of cells in G0/G1, S and G2/M cell cycle phases, and percentage of cell death and polyploidy (representative graph); (**B**) analysis of G0/G1, S and G2/M cell cycle phases. VIS light dose: 247.3 J/cm^2^.

We then analyzed the effect of VIS/wIRA irradiation with or without FB on the ability of fibroblasts to undergo the different stages of cell cycle by flow cytometry. As shown in Figure 4, incubation with FB alone (1 mg/mL) did not induce alterations in the cell cycle as compared to the control. When fibroblasts were exposed to VIS light (247.3 J/cm^2^), we observed a decreasing tendency in the percentage of cells going through G0/G1, and a tendency to increase in the percentage of cells in S and G2/M. After FB+VIS treatment, G0/G1 further decreased and S and G2/M further increased.

**Figure 5:**
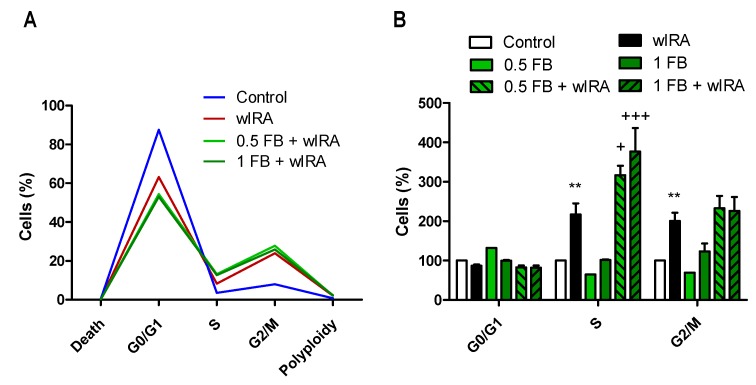
Effects of wIRA and FB+wIRA on cell cycle. Cell cycle was evaluated 24 h after treatments by flow cytometry. (**A**) Percentage of cells in G0/G1, S and G2/M cell cycle phases, and percentage of cell death and polyploidy (representative graph); (**B**) analysis of G0/G1, S and G2/M cell cycle phases. wIRA dose = 1.56 J/cm^2^. (** *p* ≤ 0.01, with respect to Control; ^+^ *p* ≤ 0.05, ^+++^ *p* ≤ 0.001, with respect to wIRA).

After wIRA exposure (1.56 J/cm^2^) (Figure 5), the percentage of cells going through G0/G1 tended to decrease and cells going through S and G2/M increased significantly (*p* ≤ 0.01) with respect to control cells. Incubation with either 0.5 or 1 mg/mL FB alone did not induce any significant changes in the cell cycle. In the case of FB+wIRA treatments, both 0.5FB+wIRA and 1FB+wIRA tended to further decrease in G0/G1 and further increase in G2/M, and significantly in S (*p* ≤ 0.05 and *p* ≤ 0.01), with respect to wIRA conditions.

### 2.3. Effects of VIS/wIRA Light and FB+VIS/wIRA Treatment on Extracellular Matrix-Related Proteins

In order to analyze the effects of VIS and wIRA lights on extracellular matrix and the potential protective role of FB, the expression of specific constituents of ECM (FBN1/2 and ELN) and enzymes involved in its remodeling (MMP-1 and CTSK) was analyzed by RT-PCR and/or immunofluorescence (IF).

#### 2.3.1. Matrix Metalloproteinase 1 (MMP-1)

**Figure 6:**
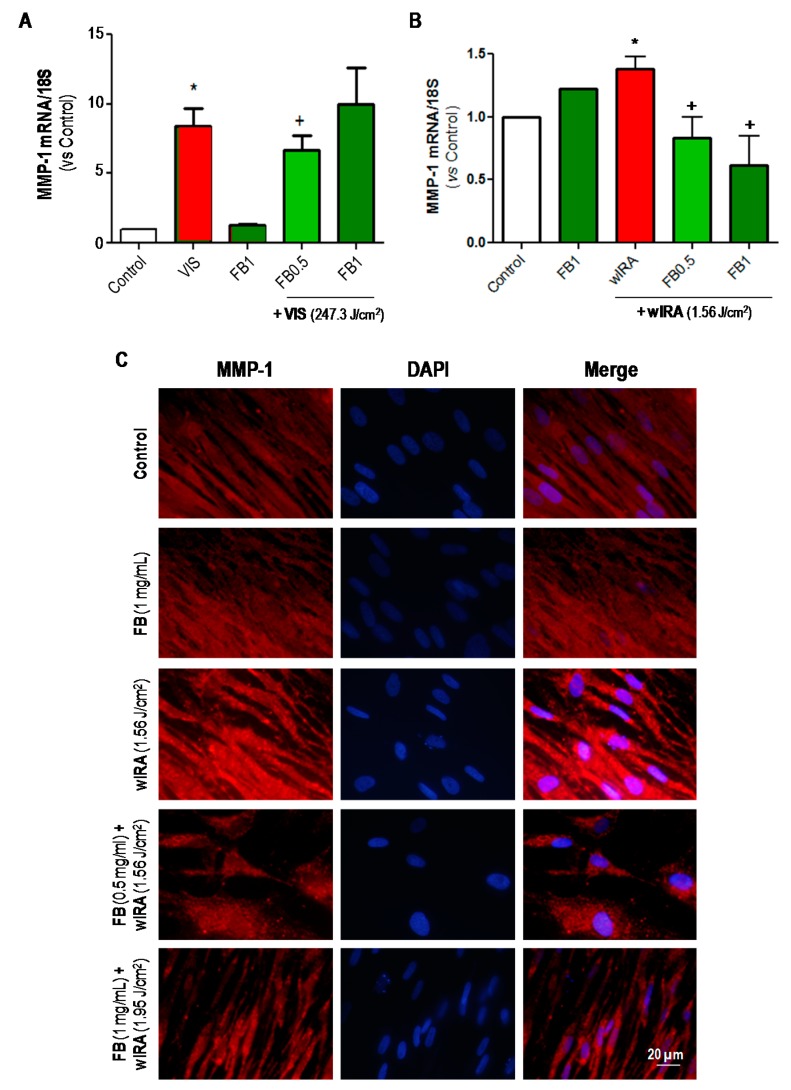
Effects of VIS/wIRA and FB+VIS/wIRA on MMP-1 expression. Cells were subjected to FB, VIS/wIRA light or FB+VIS/wIRA treatments. The expression of MMP-1 after VIS and FB+VIS treatments was quantified by RT-PCR (**A**). The expression of MMP-1 after wIRA and FB+wIRA treatments was quantified by RT-PCR (**B**) and displayed by IF (**C**). (* *p* ≤ 0.05, with respect to Control; ^+^ *p* ≤ 0.05, with respect to VIS or 1.56 J/cm^2^ wIRA). Scale bar: 20 µm.

Figure 6A shows the results from RT-PCR assay, which indicated that MMP-1 expression was significantly increased 24 h after VIS exposure (247.3 J/cm^2^) as compared to basal (control) expression. Pre- treatment with FB 0.5 mg/mL significantly decreased MMP-1 expression with respect to VIS. This was not observed with FB 1 mg/mL. In the case of wIRA light, the results from RT-PCR assay (Figure 6B) revealed a significant increase in MMP-1 expression after irradiation (1.56 J/cm^2^). Incubation with both doses of FB prior to wIRA irradiation significantly prevented this increase. These results were also qualitatively observed by IF assay (Figure 6C).

#### 2.3.2. Cathepsin K (CTSK)

**Figure 7:**
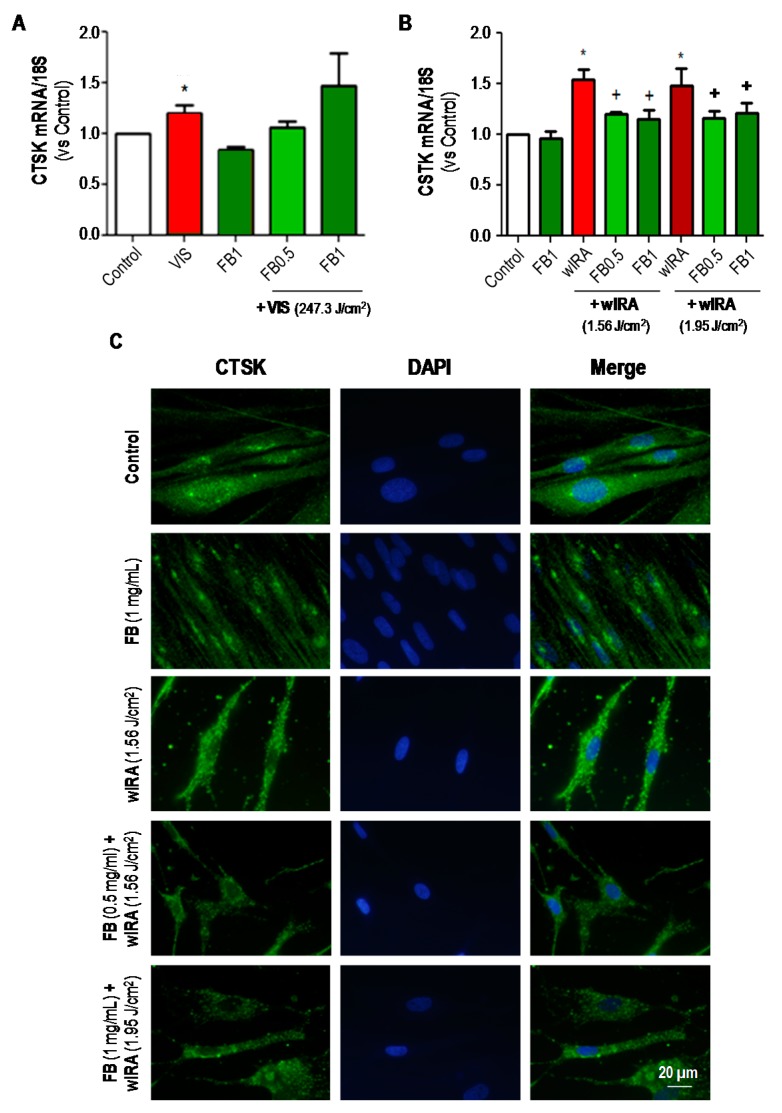
Effects of VIS/wIRA and FB+VIS/wIRA on cathepsin K (CTSK) expression. Cells were subjected to FB, VIS/wIRA light or FB+VIS/wIRA treatments. The expression of CTSK after VIS and FB+VIS treatments was quantified by RT-PCR (**A**); the expression of CTSK after wIRA and FB+wIRA treatments was quantified by RT-PCR (**B**) and displayed by IF (**C**). (* *p* ≤ 0.05, with respect to Control; ^+^ *p* ≤ 0.05, with respect to VIS or wIRA). Scale bar: 20 µm.

CTSK expression in dermal fibroblasts after VIS and FB+VIS was evaluated by RT-PCR (Figure 7A). FB per se did not induce changes in CTSK expression. Nevertheless, VIS light significantly increased the expression of CTSK (*p* ≤ 0.05), as compared to control. In the case of combined treatments, pre-treatment with FB at 0.5 mg/mL tended to decrease CTSK expression while this was not observed with FB 1 mg/mL. In the case of wIRA and FB+wIRA treatments, CTSK expression was analyzed by both RT-PCR (Figure 7B) and IF (Figure 7C). Exposure to wIRA light induced a significant increase in the expression of this marker in comparison to control (*p* ≤ 0.05). However, pre-treatment with FB (0.5 or 1 mg/mL) was able to prevent this increase, keeping CTSK expression close to the levels observed in untreated cells. Taken together, these results showed that expression of CTSK was increased in fibroblasts after exposure to both VIS and wIRA lights, and that in cells treated with FB such increase was prevented.

#### 2.3.3. Fibrillin 1 (FBN1) and Fibrillin 2 (FBN2)

**Figure 8:**
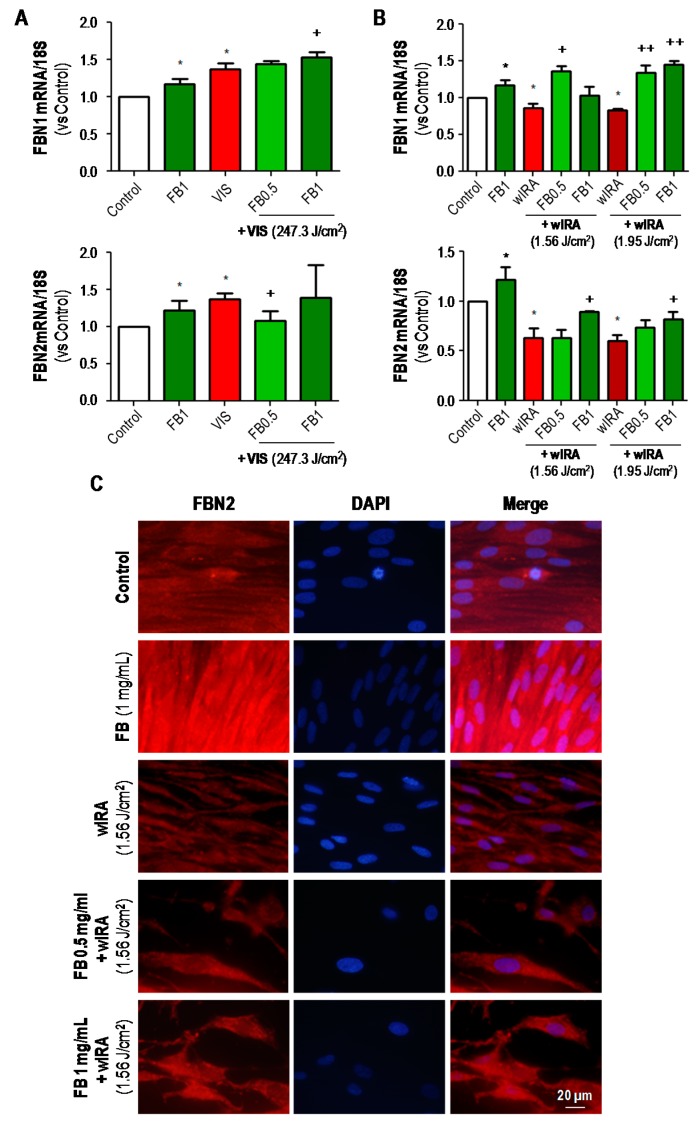
Effects of VIS/wIRA and FB+VIS/wIRA on FBN1 and FBN2 expression. Cells were subjected to FB, VIS/wIRA light or FB+VIS/wIRA treatments. The expression of FBN1 and FBN2 after VIS and FB+VIS treatments was quantified by RT-PCR (**A**); the expression of FBN1 and FBN2 after wIRA and FB+wIRA treatments was quantified by RT-PCR (**B**) and displayed by IF (**C**). (* *p* ≤ 0.05, with respect to Control; ^+^ *p* ≤ 0.05, with respect to VIS or wIRA; ^++^ *p* ≤ 0.01, with respect to VIS or wIRA). Scale bar: 20 µm.

The expression of FBN1 and FBN2 after treatments was also analyzed by RT-PCR and/or IF (Figure 8). Regarding the RT-PCR results (Figure 8A,B), it should be noted that the expression of both fibrillins was significantly increased after incubation with FB in all cases, as compared to the control. In the case of VIS treatments (Figure 8A), FBN1 expression was increased in cultures exposed to VIS light alone or in combination with FB, as compared to control cultures. Moreover, the expression of this marker was significantly higher after 1 mg/mL FB combined treatment than after VIS alone (*p* ≤ 0.05). In the case of FBN2, expression was increased after VIS alone or in combination with FB 1 mg/mL, but pre-treatment with FB 0.5 mg/mL prevented this increase. RT-PCR results for wIRA treatments are shown in Figure 8B. wIRA radiation induced a decrease in FBN1 expression. However, when wIRA exposure was preceded by incubation with FB, FBN1 expression was significantly increased (*p* ≤ 0.05 and *p* ≤ 0.01). In the case of FBN2, wIRA irradiation also decreased the expression of this protein and pretreatment with FB significantly increased the expression values. These results were also qualitatively observed by IF assay (Figure 8C).

#### 2.3.4. Elastin (ELN)

**Figure 9:**
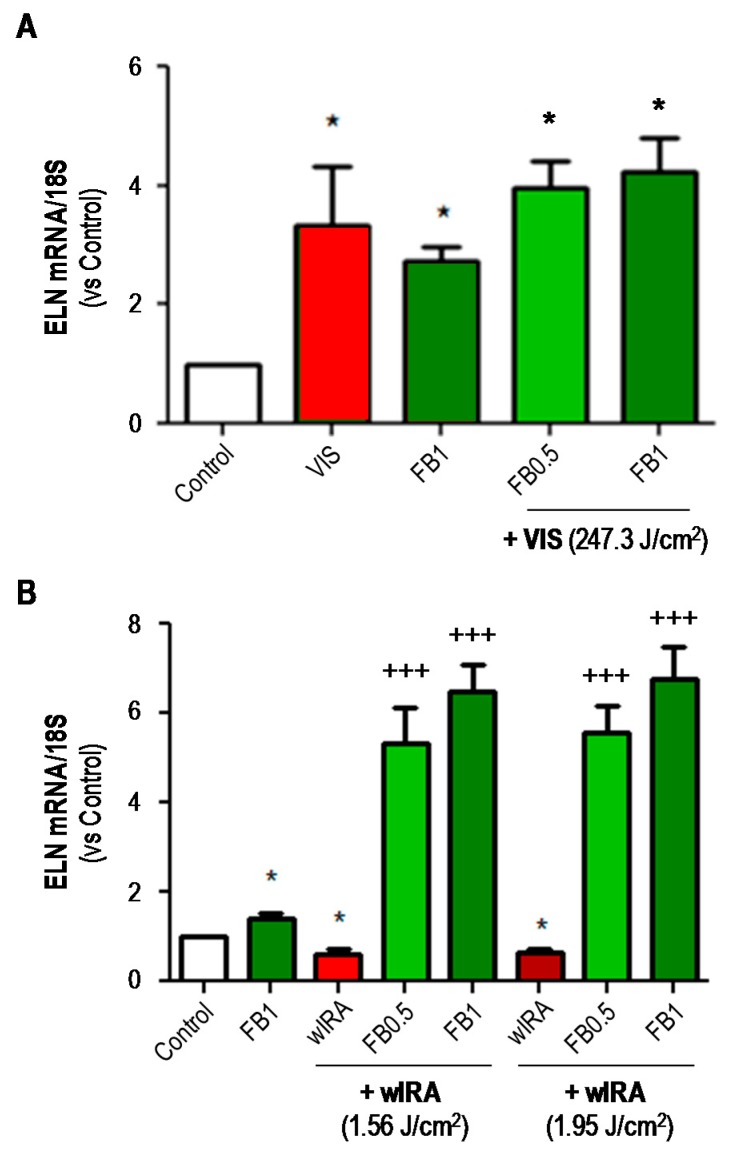
Effects of VIS/wIRA and FB+VIS/wIRA on elastin (ELN) expression. Cells were subjected to FB, VIS/wIRA light or FB+VIS/wIRA treatments. The expression of ELN after VIS or FB+VIS treatments (**A**) and after wIRA and FB+wIRA treatment (**B**) was quantified by RT-PCR. (* *p* ≤ 0.05, with respect to Control; ^+++^ *p* ≤ 0.001, with respect to wIRA).

The expression of ELN was analyzed by RT-PCR assay (Figure 9). In general, FB per se increased the expression of ELN mRNA as compared to controls. VIS irradiation also significantly increased the expression of this marker, with or without pre-treatment with FB (*p* ≤ 0.05) (Figure 9A). Conversely, wIRA irradiation induced a significant decrease in the expression of ELN, which was reversed (5 to 6-fold increase) by pre-treatment with FB (*p* ≤ 0.001) (Figure 9B).

## 3. Discussion

Extracts of various botanicals endowed with antioxidant activity are emerging as new photoprotective compounds. A natural extract from *Polypodium leucotomos*, FB, has shown antioxidant and photoprotective properties and exhibits a strong anti-aging effect. In this study, we evaluated the protective capacity of FB against the detrimental effects of IRA and VIS radiation in human dermal fibroblasts. We report that FB prevents the changes induced by wIRA and VIS on the morphology and viability of human fibroblasts, as well as on the expression of different ECM components, including MMP-1, CTSK, fibrillins 1 and 2 and elastin.

We showed here that both VIS and wIRA radiation induced damage, affecting fibroblast morphology, causing cytoplasmic retraction and cellular stretching and rounding. Moreover, exposure to both these types of radiation also increased cell death. Previous studies have revealed the ability of *P. leucotomos* to preserve human fibroblasts after UVA light exposure [25] and to prevent the epidermal cellular damage caused by UVB light [26]. In this work we wanted to explore whether *P. leucotomos* extract was also able to prevent cell damage caused by VIS and wIRA radiation. Even though FB did not prevent the morphological alterations caused by irradiation, results suggested that it played a role in preventing cell death. We used two FB doses, 0.5 and 1 mg/mL in our experiments. Of the two doses, 0.5 mg/mL seems to generally produce more consistent and sometimes more favorable results. We believe this might be due to achieving a maximal dose-effect with 0.5 mg/mL in our experimental conditions. Results on cell cycle stages suggested that VIS, and especially wIRA radiation affected the percentage of fibroblasts found at specific points throughout cell cycle. The observed decrease of cells in the G0/G1 phase and the correlated increase of cells in the S and G2/M phases (mitotic arrest) could be the cellular light-induced response prior to the onset of a cell death program [27,28]. As results indicated, treatment with FB did not return results to control levels. Since FB is non-toxic, that is, it does not increase cell death nor cell proliferation, it might increase the percentage of cells in the S phase to allow for damaged DNA to be repaired. This effect has previously been described for ferulic acid in human dermal fibroblasts subjected to UVA radiation [29].

Dermal fibroblasts constitute the major cell type in the dermis and have historically been considered the main contributors responsible for the synthesis and remodeling of extracellular matrix [30]. In order to analyze the effects of VIS and wIRA light on extracellular matrix and the potentially protective role of FB, the expression of specific constituents of ECM (FBN1/2 and ELN) and enzymes involved in its remodeling (MMP-1 and CTSK) was analyzed. Matrix metalloproteinases (MMPs) are a family of collagenolytic enzymes involved in different physiological and pathological processes. They regulate several signaling pathways in cell growth, inflammation and angiogenesis and are able to degrade the components of extracellular matrix. Specifically, MMP-1 has collagenase activity and breaks down the interstitial collagen I, II and II, yielding denatured collagen (gelatin) [9]. A previous study carried out in human fibroblasts revealed that *P. leucotomos* inhibits MMP-1 expression in UVB or UVA-exposed human fibroblasts [21]. Moreover, *P. leucotomos* also stimulates the expression of TIMPs in UVB or UVA-exposed epidermal keratinocytes [31]. The fact that FB has a similar effect also at VIS and wIRA wavelengths, suggests that FB’s effect on curbing the expression of matrix degradation enzymes does not depend on the wavelengths of the triggering stimulus.

Cathepsins are a group of cysteine proteinases that are involved in various aspects of extracellular matrix remodeling. Collagenolytic activity of CTSK plays a pivotal role in bone resorption and lung matrix homeostasis. CTSK has also been reported to be expressed in other tissues. In the dermis, it is expressed only under certain circumstances such as scarring or inflammation [24,32]. Our results showed that expression of CTSK increased in fibroblasts after exposure to both VIS and wIRA light. FB was able to prevent such increase, indicating that CTSK could be an important factor during photoaging that is curbed by FB.

Fibrillins 1 and 2 (FBN1 and FBN2) are the main constituents of the extracellular microfibrils responsible for the biomechanical properties of most tissues and organs. They also play an important role in the assembly of elastin during the formation of elastic fibers. FBN1 provides mostly force-bearing structural support, whereas FBN2 predominantly regulates the early process of elastic fiber assembly [22]. Our results indicate that FB per se stimulated the expression of FBN1 and FBN2. On the other hand, VIS and wIRA light exerts an opposite effect on the expression of these fibrillins; while VIS caused an increase on their expression, wIRA induced a decrease. After FB treatment though, both FBN1 and FBN2 expression was generally increased after either VIS or wIRA radiation. These results are in line with those reported in 2009 by Philips et al. [31], where UV light had an inhibitory effect on fibrillins of keratinocytes, and *P. leucotomos* stimulated the expression of fibrillins in both non-UV-exposed and UV-exposed keratinocytes.

Elastin (ELN) is a major component of ECM. Reduced elastin levels are associated with various diseases such as atherosclerosis and arthritis. Among the changes that affect cutaneous tissue with age, the loss of elastic properties caused by increased elastin degradation and/or processing and changes in elastin production cause a substantial impact on tissue esthetics and health. The occurrence of solar elastosis is one of the main markers of cutaneous photoaging and is characterized by disorganized and non-functional deposition of elastic fibers [33]. In summary, as with fibrillins, our results indicate that FB per se promoted the expression of ELN in fibroblasts. VIS light stimulated the expression of this marker while wIRA inhibited it. However, when irradiation was preceded by FB treatment, ELN was markedly increased, especially in the case of wIRA. This correlates well with previous observations by Philips et al. [31] describing the inhibitory effect of *P. leucotomos* on keratinocyte elastase activity.

We report that FB prevents the changes induced by wIRA and VIS on the morphology and viability of human fibroblasts, as well as on the expression of different ECM components including MMP-1, CTSK, fibrillins 1 and 2 and elastin. Overall, our results suggest that FB could be a preventive treatment against skin cellular damage and ECM alterations caused by VIS and wIRA radiation, and thus could be beneficial in preventing photoaging.

## 4. Materials and Methods

### 4.1. Reagents

FB, a controlled hydrophilic extract from the leaves of *Polypodium leucotomos*, was obtained as a lyophilized powder from Cantabria Labs, Madrid, Spain. The methods for obtaining the extract have been previously published [34]. The extract was stored at room temperature and shielded from light following the provider’s instructions, and then a stock solution was prepared at a concentration of 10 mg/mL in distilled water, under agitation at 25–30 °C. This stock was diluted in the culture medium to the desired concentration. Previous studies already published by the group have tested the efficacy of the extract against UV light at concentrations ranging from 0.01 through to 10 mg/mL [19,21,35]. Based on those studies, we determined to treat fibroblasts with 0.5 and 1 mg/mL of FB for 24 h before irradiation.

### 4.2. Cell Cultures

Human dermal fibroblasts (HDF) were obtained from a skin biopsy after two rounds of trypsinization with 0.25% Trypsin-EDTA by shaking and at 37 °C. HDF were cultured in Dulbecco’s modified eagle medium (DMEM) supplemented with 10% (*v*/*v*) fetal bovine serum (FBS) and 1% (*v*/*v*) penicillin G (100 U/mL) and streptomycin (100 µg/mL) (HyClone Laboratories, South Logan, UT, USA). Cells were maintained at 37 °C, 5% humidity and 5% CO_2_ in an incubator (Heraeus HERAcell, Thermo Scientific, Waltham, MA, USA).

### 4.3. Irradiation

Fibroblasts were subjected to VIS or wIRA radiation. The source of VIS was a 400–700 nm lamp (White 4500 K, Segainvex, Madrid, Spain) (Figure S1A). The infrared radiation source was an infrared-A lamp with water filter and OG590 orange filter (550–1400 mm, Hydrosun^®^, Müllheim, Germany) (Figure S1B). In order to evaluate the cellular damage induced by the radiation, VIS and wIRA light doses were tested (61.8–247.3 J/cm^2^ in the case of VIS and 0.78–1.95 J/cm^2^ in the case of wIRA). According to the harmful effects observed after light exposure, we selected doses of 247.3 J/cm^2^ in the case of VIS and 1.56 or 1.95 J/cm^2^ in the case of wIRA to carry out the combined treatments (incubation with FB + irradiation). Immediately after irradiation, fibroblasts were maintained in the incubator for 24–48 h before processing.

### 4.4. Cell Morphology Analysis

Cell cultures were observed in vivo at different time points (0, 24 and 48 h) after FB+VIS/wIRA treatments using an inverted microscope (Olympus IX51, Olympus Surgical Technologies Europe, Hamburg, Germany). Morphological changes were qualitatively analyzed from captured images.

### 4.5. Immunofluorescence Assays

Fibroblasts were seeded on glass coverslips (Menzel-Gläser, Braunschweig, Germany) and when they reached an appropriate confluence (approximately 80%), they were incubated with FB (0.5 or 1 mg/mL) for 24 h. Cells were then washed with PBS and irradiated with wIRA light. After treatment, cells were fixed with 3.7% formaldehyde (Panreac, Barcelona, Spain) and then permeabilized with 0.01% Triton X-100 in PBS before incubation with specific primary antibodies for matrix metalloproteinase-1 (MMP-1), cathepsin K (CTSK) and fibrillin 2 (FBN2) (Table S1). Afterwards, coverslips were incubated with specific anti-IgG secondary antibodies coupled to AlexaFluor488 or AlexaFluor546 (Table S1). Finally, coverslips were mounted on slides using ProLong^®^ Gold Antifade Mountant medium containing DAPI (Thermo Fisher Scientific, Waltham, MA, USA).

### 4.6. Determination of Cell Survival/Proliferation

Cell survival was assessed by flow cytometry. In order to quantify the cell death rate, cells were trypsinized 24 h after FB + VIS or FB + wIRA treatments, collected, incubated with propidium iodide for 5 min and analyzed using a flow cytometer (Cytomics FC 500, Beckman Coulter, Brea, CA, USA).

### 4.7. Analysis of Cell Cycle

Near confluent cell monolayers were tripsinized 24 h after treatment (FB+VIS or FB+wIRA) and centrifuged for 5 min (1800 rpm). Cells were then processed using a DNAprep kit (Qiagen, Hilden, Germany) according to manufacturer’s recommendations. Finally, samples were evaluated by flow cytometry.

### 4.8. Analysis of mRNA Expression

Fibroblast cultures were homogenized 24 h after treatment (FB+VIS or FB+wIRA) and mRNA was isolated by RNeasy kit (Qiagen, Hilden, Germany). mRNA concentration and its quality (A260:A280 ratio ≥ 1.8) were checked by spectrophotometry (NanoDropND1000, Nanodrop Technologies, Wilmington, DE, USA). The mRNA expression was analyzed by RT-PCR (Reverse Transcription Polymerase Chain Reaction), using a RT-PCR kit (Roche) and specific primers for MMP-1, fibrillin 1, fibrillin 2 and elastin (Table S2). Semi-quantitative analysis of RT-PCR results was performed using DDCt method (Light Cycler^®^ 480 Software, version 1.5, Roche Molecular Systems, Indianapolis, IN, USA), which corrects the data obtained from each sample relative to rRNA 18S reference value from each cDNA. Results were transformed into RQ (relative quantity) values using the control samples for normalization (RA = 1.00). All RT-PCR analyses were carried out at Genomics Unit, Parque Científico de Madrid, Madrid, Spain.

### 4.9. Image Analysis

Microscopic images were obtained using an epifluorescence microscope coupled to a CCD camera DP70 (Olympus BX-61, Tokyo, Japan) with UV filters for the excitation light (360–370 nm excitation filter UG-1), blue (450–490 nm excitation filter BP 490), or green (570–590 nm excitation filter 590 DM). Picture processing was performed with Photoshop Extended CS5 12.0 (Adobe Systems Inc., Mountain View, CA, USA).

### 4.10. Statistical Analysis

Data are represented as the mean ± standard deviation (SD) of at least three independent experiments. Statistical significance was determined by statistical test of analysis of variance (ANOVA) or two-way ANOVA and Bonferroni post hoc tests, using GraphPad Prism 5.00 (GraphPad Software, Inc., San Diego, CA, USA). Differences were considered to be significant when *p* ≤ 0.05.

## Supplementary Materials

Supplementary materials can be found at http://www.mdpi.com/1422-0067/19/8/2250/s1.

## Abbreviations

ANOVA	Analysis of variance
AP-1	Activator protein 1
COX-2	Cyclooxygenase-2
CTSK	Cathepsin K
DMEM	Dulbecco’s modified eagle medium
ECM	Extracellular matrix
FACS	Fluorescent-activated cell sorting
FB	Fernblock^®^
HDF	Human dermal fibroblasts
IF	Immunofluorescence
IPD	Immediate pigment darkening
IR	Infrared
MAPKs	Mitogen-Activated Protein Kinases
MMPs	Matrix metalloproteinases
NFκB	Nuclear factor kappa-light-chain-enhancer of activated B cells
PL	*Polypodium leucotomos*
PPD	Persistent pigment darkening
ROS	Reactive oxygen species
SD	Standard deviation
TIMPs	Tissue inhibitors of metalloproteases
UV	Ultraviolet light
VIS	Visible light
wIRA	Water-filtered Infrared A
