# Supplementary material for: Fernblock Prevents Dermal Cell Damage Induced by Visible and Infrared A Radiation
Source: Int J Mol Sci. 2018 Aug 1;19(8):2250. doi: 10.3390/ijms19082250 (PMC6121512; doi:10.3390/ijms19082250)
Supplement: Supplementary file 1 [file ijms-19-02250-s001.pdf]

SUPPLEMENTARY MATERIAL

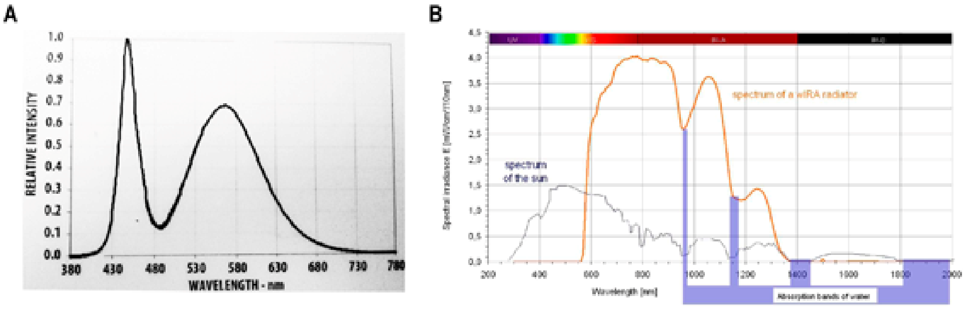

Figure S1. Spectra of light sources. Power and wavelength range of VIS (**A**) and wIRA (**B**) sources.

Antigen	Origin	Source	Dilution
Matrix metalloproteinase 1 (MMP-1)	Rabbit (monoclonal)	Abcam®	1:100
Cathepsin k	Rabbit (polyclonal)	Sigma®	1:100
Fibrillin 2	Mouse (monoclonal)	Novus biologicals®	1:100
Mouse IgG -Alexa 488	Goat (polyclonal)	Invitrogen®	1:250
Rabbit IgG-Alexa 546	Goat (monoclonal)	Invitrogen®	1:250

Table S1. Antibodies used for immunofluorescence. Antigen, origin, source and working dilution of primary and secondary antibodies used to perform IF assays.

Gen	Species	Forward	Reverse
MMP-1	Human	CATGAAAGGTGGACCAACAATTT	CCAAGAGAATGGCCGAGTTC
Cathepsin k	Human	CTTTGCTCTGTACCCTGAGGAGAT	TGTTATATTGCTTCCTGTGGG TCT T
Fibrillin 1	Human	TGACTGGCCCACACGTGCATAG	TGACATTGACCCCTTGTTGACAGGA
Fibrillin 2	Human	GGACATTGACGGATGTGA	GATGCCGTTTCCAATCCA
Elastin	Human	CTAAATACGGTGCTGCTGGC	CATGGGATGGGGTTACAAAG

Table S2. Sequences of primers used for mRNA analysis. Gen, species and forward/reverse sequences.
